# Whole exome sequencing identifies two novel variants in *PHEX* and *DMP1* in Malaysian children with hypophosphatemic rickets

**DOI:** 10.1186/s13052-022-01385-5

**Published:** 2022-12-08

**Authors:** Nahid Tavana, Tzer Hwu Ting, Kaitao Lai, Marina L. Kennerson, Karuppiah Thilakavathy

**Affiliations:** 1 Department of Biomedical Science, Faculty of Medicine and Health Sciences, Universiti Putra Malaysia, UPM Serdang, Selangor Malaysia; 2 Department of Paediatrics, Faculty of Medicine and Health Sciences, Universiti Putra Malaysia, UPM Serdang, Selangor Malaysia; 3 Northcott Neuroscience Laboratory, ANZAC Research Institute, University of Sydney, Concord, NSW Australia; 4 Sydney Medical School, University of Sydney, Sydney, NSW Australia; 5 Molecular Medicine Laboratory, Concord Hospital, Concord, NSW Australia; 6 Genetics and Regenerative Medicine Research Group, Faculty of Medicine and Health Sciences, Universiti Putra Malaysia, UPM Serdang, Selangor Malaysia

**Keywords:** Hypophosphatemic rickets, Phosphate, *PHEX*, *DMP1*, Mutation, Whole exome sequencing, Novel

## Abstract

**Background:**

Hypophosphatemic rickets (HR) is a genetic disease of phosphate wasting that is characterized by defective bone mineralization. The most common cause of the disease is mutations in the phosphate regulating gene with homologies to endopeptidases on the X chromosome (*PHEX*) gene. The aims of this study were to identify the gene variants responsible for HR in three cases of Malaysian origin from three independent families and to describe their clinical, biochemical, and radiological features.

**Methods:**

Whole exome sequencing (WES) was performed on all patients and their parents, followed by Sanger sequencing validation. Bioinformatics tools were used to provide supporting evidence for pathogenicity of variants. To confirm that a mutation is de novo, paternity test was carried out. High resolution melting curve analysis was performed to assess the allele frequency in normal controls for mutations that were found in the patients.

**Results:**

The patients showed typical characteristics of HR including lower limb deformity, hypophosphatemia, and elevated alkaline phosphatase. WES revealed two variants in the *PHEX* gene and one variant in the dentin matrix protein 1 (*DMP1*) gene. Two of the three variants were novel, including c.1946_1954del (p.Gly649_Arg651del) in *PHEX* and c.54 + 1G > A in *DMP1*. Our data suggests that the novel p.Gly649_Arg651del variant is likely pathogenic for HR disease.

**Conclusions:**

This study extends the variant spectrum of the *PHEX* and *DMP1* genes. Our findings indicate that WES is an advantageous approach for diagnosis of genetic diseases which are heterogeneous.

**Supplementary Information:**

The online version contains supplementary material available at 10.1186/s13052-022-01385-5.

## Background

Hypophosphatemic rickets (HR) is a genetic disorder in which the proximal renal tubule cannot reabsorb sufficient phosphate (Pi), leading to growth retardation, rickets, and osteomalacia [1]. The most frequent form of hereditary HR is X-linked hypophosphatemic rickets (XLHR; MIM#307800), in which 80% of familial HR and 70% of sporadic HR are diagnosed as XLHR [2]. XLHR is caused by loss-of-function (LOF) mutations in the phosphate regulating gene with homologies to endopeptidases on the X chromosome (*PHEX*).

The XLHR inheritance pattern is X-linked dominant as both males and females present with the disease, and its prevalence is 1 in 20,000 live births [3]. The number of *PHEX* mutations reported in the Human Gene Mutation Database (HGMD) [4] is more than 400 mutations, most of which are missense and nonsense mutations, however other types including small deletions/insertions, abnormal splicing, and gross insertions/deletions have also been reported [1, 5].

Other less common forms of hereditary HR include autosomal dominant HR (ADHR), autosomal recessive HR 1 (ARHR1), autosomal recessive HR 2 (ARHR2), hereditary HR with hypercalciuria (HHRH), and X-linked recessive HR (XRHR) which are caused by gene mutations in fibroblast growth factor 23 (*FGF23),* dentin matrix acidic phosphoprotein 1 (*DMP1*)*,* ectonucleotide pyrophosphatase/phosphodiesterase 1 (*ENPP1*), solute carrier family 34, member 3 (*SLC34A3*)*,* and chloride channel 5 (*CLCN5*), respectively.

FGF23 plays an important role in regulating phosphate and vitamin D homeostasis. PHEX, DMP1 and ENPP1 negatively regulate the expression of FGF23, which is produced in osteocytes and secreted into the circulation [6, 7]. The deficiencies of these genes lead to excessive secretion of FGF23. Excess FGF23 causes hypophosphatemia in two ways. The expression of phosphate co-transporters in the kidney is suppressed by FGF23, leading to decreased phosphate reabsorption. FGF23 also reduces the production of 1,25-dihydroxyvitamin D (1,25(OH)_2_D3) and thus decreases the absorption of phosphate in the intestine. Other biochemical findings include elevated serum alkaline phosphatase (ALP) and normal to slightly high parathyroid hormone (PTH) levels [8, 9].

The clinical features are similar in various forms of hypophosphatemia, although this does not imply that the symptoms are identical. Children mostly present with rickets, short stature, dental abscesses, and lower limb bone deformities. Symptoms of the disease in adults include osteomalacia, bone pain, and enthesopathy [5]. Treatment for XLHR, ADHR or ARHR, includes oral phosphate and vitamin D supplementations while, patients with HHRH are only treated with phosphate supplementation [10]. This conventional treatment improves rickets in children. However, a significant number of patients may not show successful results [11] and complications such as nephrocalcinosis and hyperparathyroidism have been reported in some cases [12]. In addition to the conventional treatments, burosumab, which was approved by the US Food and Drug Administration in 2018, is used to treat XLHR. Burosumab is a human monoclonal antibody targeting FGF23, thus, significantly improves rickets, growth, and biochemical abnormalities [8, 12].

The rarity of HR may lead to delayed diagnosis. In addition, the recent developed treatment for this disease cannot be prescribed to the patients without defining and accurately diagnosing the disease. Therefore, correct and early diagnosis of this disease is important for optimal treatment. In the Malaysian population, there is very little information about the clinical, biochemical and, most importantly, genetic features of HR [13, 14]. Previously, only one genetic study of HR disease had been performed in Malaysia in which Sanger sequencing for *PHEX*, *FGF23* and *DMP1* genes in four Malaysian HR pediatric patients revealed two *PHEX* variants [14].

In the present study, we report clinical, radiological, laboratory, and genetic data of three pediatric patients with HR in Malaysia, for which whole exome sequencing (WES) revealed three candidate pathogenic variants. Further analysis was employed to support the pathogenicity of the variants.

## Methods

### Subjects and study approval

Pediatric patients with HR were identified from the pediatric endocrine clinic at Hospital Serdang and Hospital Kuala Lumpur, Malaysia in 2019. The inclusion criteria for the study were patients presenting with clinical and biochemical features of HR including lower limb bowing deformity, short stature, low serum phosphate, elevated alkaline phosphatase, normal serum calcium, and normal 25-hydroxyvitamin D. Informed consent for genetic investigation was obtained from the patients’ parents. Peripheral whole blood samples from three patient-parent trios were collected in lithium heparin-containing tubes and genomic DNA (gDNA) was extracted using the QIAamp DNA Blood Midi Kit (QIAGEN, Hilden, Germany). Approval for ethics was obtained from the Medical Research and Ethics Committee (MREC), Ministry of Health, Malaysia (reference number: NMRR-17-612-33,842 (IIR)).

### Genetic analysis

The DNA samples of three affected children and their parents from unrelated families were sent to Macrogen (Seoul, South Korea) for WES. Sequencing libraries were generated using SureSelect V5-post kit (Agilent Tech. Ltd., USA) with 1 μg of gDNA. The size of the PCR-enriched fragments was verified by testing the template size distribution on the 2100 Bioanalyzer (Agilent Tech. Ltd., USA) using a DNA 1000 chip. Quantification of DNA libraries was done using a qPCR technique based on the Illumina qPCR Quantification Protocol Guide. The captured DNA was sequenced as paired end reads (150 bp) on an Illumina Novaseq6000 (Illumina, CA, USA) sequencer. The paired-end sequences were aligned to the human reference genome assembly GRCh37 (hg19) using the Burrows-Wheeler Aligner (BWA) software. Duplicate reads were removed using Picard software. Sequence variant calling was performed using Genome Analysis Tool kit (GATK) version GATK v4.0.5.1 and annotated with effect prediction using the SnpEff tool. Screening of study subjects initially involved examining the WES data for variants in the hereditary HR-related genes including *PHEX* (NM_000444.5), *FGF23* (NM_020638.2), *DMP1* (NM_004407.3), *ENPP1* (NM_006208.2), *SLC34A3* (NM_001177316.1), and *CLCN5* (NM_001127898.3). The candidate variants identified through WES were validated by Sanger sequencing using the DNA sequencing service provided by Apical (Apical Scientific Sdn. Bhd., Malaysia). The details of PCR protocol are provided in Additional file 1 Supplementary Table 1. The segregation of variants with disease phenotype in each patient-parent trio was evaluated. Since the parents in the three trios were phenotypically normal, two strategies were used to filter the variants in each trio; de novo *strategy* to find the de novo variants in affected child, and *double-hit strategy* to identify homozygous or compound heterozygous variants in affected child, recessively inherited from unaffected parents [15]. Paternity testing was performed for the patient when the parents were not carriers of the variant and there was no family history of the disease, in order to confirm the de novo occurrence of a variant. To do so, Galaxy browser [16] was used to filter the WES data for 50 SNPs which the panel has been developed and validated by Yousefi et al. [17] for the unique identification of an individual. Then, 50 normal female controls of Malay ethnicity were screened for the novel variants using HRM analysis using a LightCycler 480 instrument (Roche, Switzerland) as outlined in Additional file 1 Supplementary Table 2.

### Bioinformatics analysis of novel sequence variants

Novel variants were assessed for their allele frequency in the population databases including 1000 Genome Project [18], Genome Aggregation Database (gnomAD) [19], and Exome Sequencing Project (ESP) [20]. Moreover, the bioinformatic tools were used for novel variants to predict their impact and likelihood of causing disease. The effect of in-frame deletion was evaluated using MutationTaster [21] and MutPred-Indel [22]. To predict the impact of splicing variant, Human Splicing Finder (HSF) 3.1 [23], MaxEntScan [24], SpliceAI [25], and NNSPLICE 0.9 [26] were used. Conservation analysis of nucleotides was performed using three programs including PhyloP [27], GERP++ [28], and PhastCons [29] through the UCSC Genome Browser.

## Results

### Patients’ characteristics

**Fig. 1:**
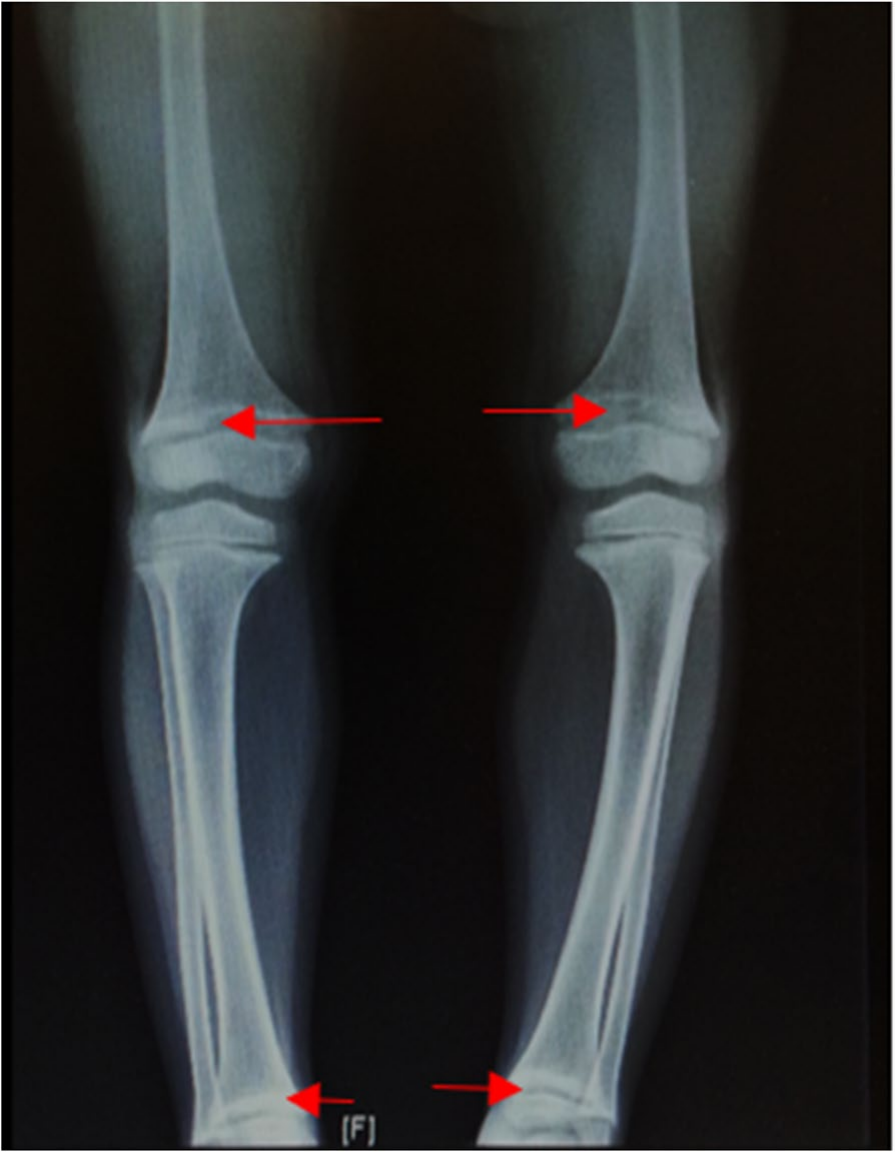
Radiograph of lower limbs of patient 1. Radiograph shows features of rickets with fraying and splaying of the metaphyses of the distal femur and distal tibia (red arrows)

There were three patients of Malay ethnicity identified and recruited for the study. Detailed clinical, biochemical, radiological, and genetic findings are summarized in Table 1. Patient 1 had delayed presentation and diagnosis of HR at 6 years. However, history taking revealed that patient 1 had bowing of legs since 1–2 years of age but the parents did not seek medical attention then. The age of onset of skeletal abnormalities for all patients in this study ranged from 1 to 2 years. Radiographs of all patients showed evidence of rickets with fraying and splaying of the metaphyses of long bones of the limbs (Fig. 1). None of the patients had family members with HR or skeletal deformity. Patient 1 and patient 2 had non-consanguineous parents, while the parents of patient 3 were consanguineous and shared the same maternal grandfather.

**Table 1:** Clinical, biochemical, radiological, and genetic findings of patients with HR

Subject	Patient 1	Patient 2	Patient 3
Gender	Female	Female	Female
Gene, Variant	*PHEX,* c.871C > T	*PHEX,* c.1946_1954del	*DMP1,* c.54 + 1G > A
Age at diagnosis (years)	6	1 year and 4 months	2
Weight (kg), percentile at diagnosis	20.5, <P3	7.3, <P3	N/A, P25–50
Height (cm), percentile at diagnosis	104, P50	70, <P3	N/A, P25–50
Growth retardation	Yes	Yes	No
Bowing of legs	Yes	Yes	Yes
Frontal bossing	Yes	Yes	No
Wrist swelling	No	Yes	Yes
Ankle swelling	No	No	No
Serum phosphate (mmol/L) Normal range: 1.1–1.8	0.78	0.52	0.81
Serum calcium (mmol/L) Normal range: 2.10–2.55	2.16	2.38	2.12
Serum ALP (U/L) Normal range: 100–350	436	1479	501
25-hydroxyvitamin-D (nmol/L) Normal range: > 50	56.5	51.0	normal^a^
Urine calcium/creatinine ratio Normal range: < 0.7	N/A	0.04	0.45
TmP/GFR Normal range: 1.07–2.23	N/A	0.51	N/A
Radiological changes	Features of rickets at distal femur and tibia	Features of rickets at distal ulna and radius	Features of rickets at distal femur and tibia, distal ulna and radius.

*ALP* alkaline phosphatase; *TmP/GFR* ratio of tubular maximum reabsorption rate of phosphate to glomerular filtration rate; *P* percentile on National Center for Health Statistics growth chart; *N/A* not available

^a^Value was not stated in the medical record and was indicated as normal

### Variants identified through whole exome sequencing and sanger sequencing confirmation

**Fig. 2:**
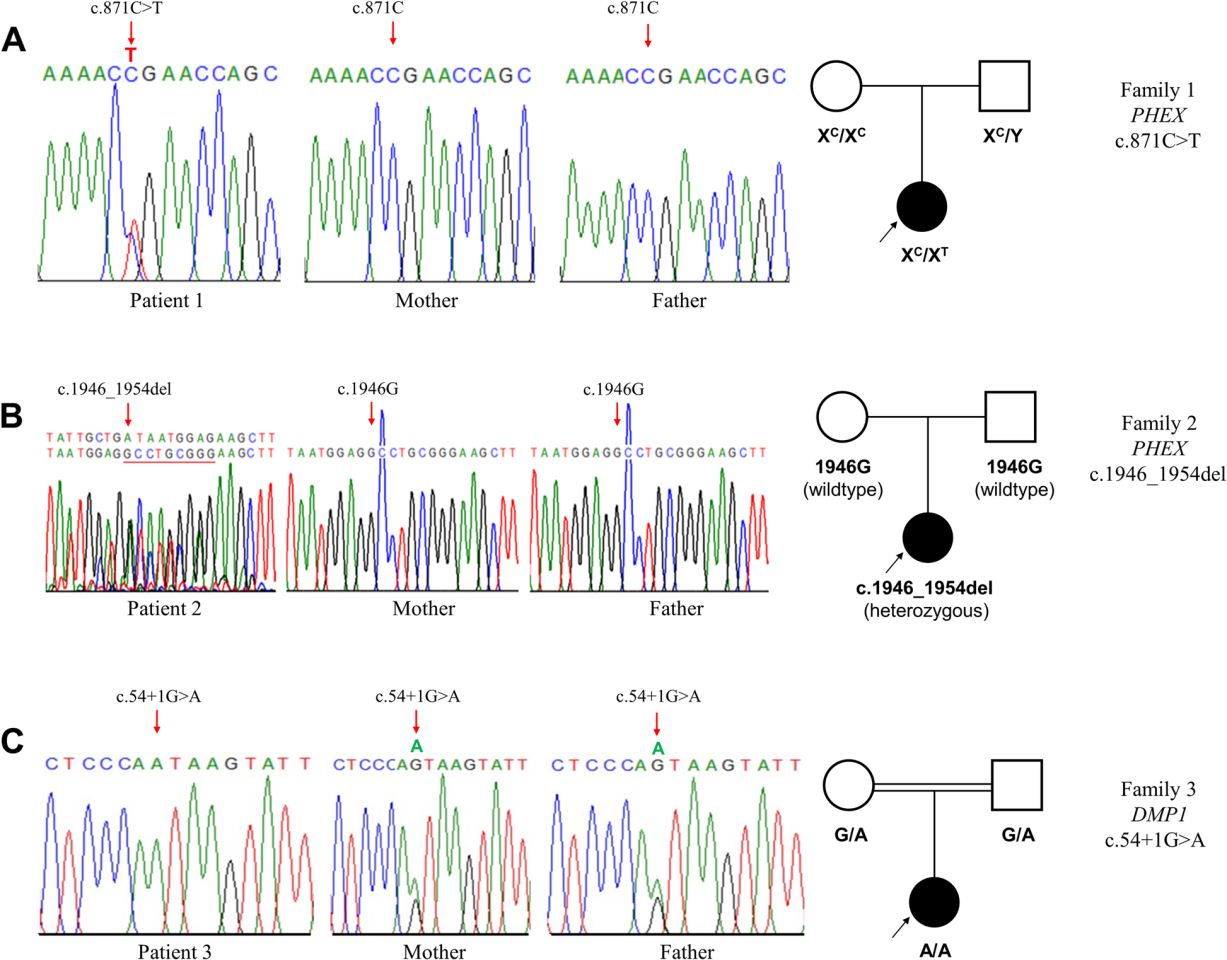
Sequence confirmation of variants and pedigrees of three families with HR. (A) Heterozygous c.871C > T variant in exon 8 of the *PHEX* gene in patient 1 and wildtype genotype in her parents. (B) Heterozygous c.1946_1954del variant in exon 19 of the *PHEX* gene in patient 2 and wildtype genotype in her parents. (C) Homozygous c.54 + 1G > A variant in intron 2 of the *DMP1* gene in patient 3 and heterozygous genotype in her parents. Circles and squares in pedigrees represent females and males, respectively. Solid circles with arrow indicate index cases. Double lines between symbols indicate consanguineous marriage. Genotype of each individual is shown under the symbols

Three parent-child trios from three unrelated families underwent WES to screen HR genes for mutations. After selecting the de novo variants and variants segregating as recessive traits, three candidate variants were identified which were absent from population databases including 1000 Genome Project, gnomAD, and ESP. A heterozygous variant in *PHEX*, c.871C > T (p.Arg291Ter), was found in patient 1 which has been reported in HGMD and ClinVar [30] and was absent in both of the patients parents. The other two variants which are novel and unreported, include the *PHEX* variant c.1946_1954del (p.Gly649_Arg651del) in patient 2 and the *DMP1* variant c.54 + 1G > A in patient 3. Sanger sequencing confirmed the presence of the three variants in each patient (Fig. 2). The likelihood of pathogenicity of the novel variants was further assessed.

### Pathogenicity analysis of novel variants

**Fig. 3:**
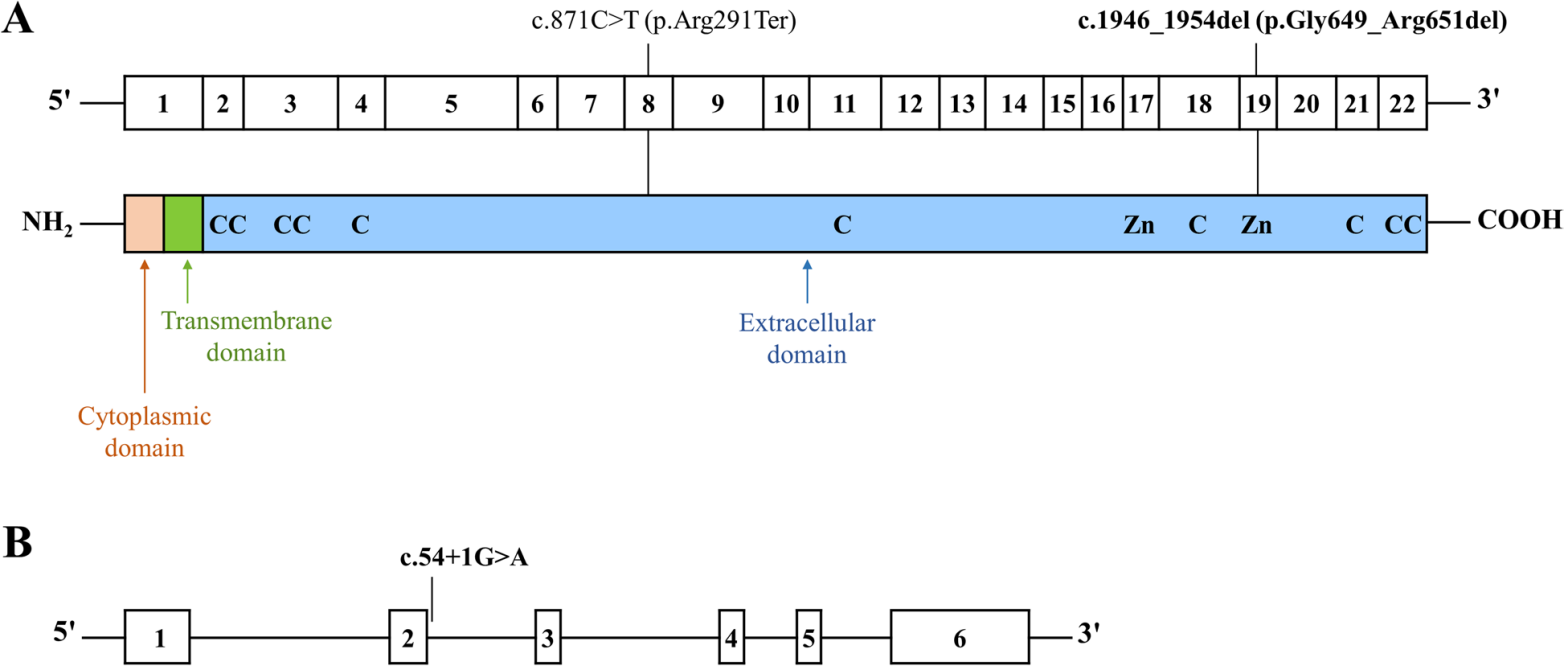
Location of *PHEX* and *DMP1* variants identified in the present study. (A) The bar with numbers represents the 22 exons of the *PHEX* cDNA. Diagram of the PHEX protein shows the three domains including the N-terminal cytoplasmic domain, the transmembrane domain, and the extracellular domain with conserved cysteine residues (C) and the zinc-binding motifs (Zn). (B) Schematic representation of the *DMP1* gene. The numbers represent the 6 exons of the *DMP1* gene. The two novel *PHEX* and *DMP1* variants are in bold

The pathogenicity of the variants was assigned according to guidelines of the American College of Medical Genetics and Genomics (ACMG) [31]. WES in this study detected one previously reported variant and two novel variants in Malay patients with HR disease. Patient 1 was heterozygous for the previously reported nonsense *PHEX* variant (c.871C > T; p.Arg291Ter) which is located in exon 8 of the gene and the extracellular domain of the PHEX protein (Fig. 3a). The variant was not inherited from her healthy parents (Fig. 2a) and was therefore considered a de novo variant with the confirmation of paternity. The p.Arg291Ter variant is reported in ClinVar as pathogenic with multiple submissions and the collective evidence supports that p.Arg291Ter in the *PHEX* gene is a pathogenic variant for HR disease (Additional file 1 Supplementary Table 3).

A novel de novo variant causing an in-frame deletion in exon 19 of the *PHEX* gene (c.1946_1954del; p.Gly649_Arg651del) was identified in patient 2 who was heterozygous for this variant. The variant was absent in both parents (Fig. 2b) and the paternity testing confirmed de novo occurrence of p.Gly649_Arg651del in this patient. Annotation of the deletion was absent in disease and population databases including Clinvar, HGMD, 1000 genome, gnomAD, and ESP. Furthermore, the variant was absent in 100 normal control chromosomes of Malay ethnicity (Additional file 1 Supplementary Fig. 1). The bioinformatic tools used for the prediction of in-frame deletion, did not predict a damaging effect on the PHEX protein. Nevertheless, the deleted positions are conserved across species (Additional file 1 Supplementary Table 3). In particular, the glycine residue 649 is highly conserved in the members of a family of zinc-metallopeptidases including the PHEX protein and is located in the zinc-binding motif of PHEX which is important for the catalytic activity [32] (Fig. 3a). Furthermore, missense variants at positions 649 and 651 of the PHEX protein have been reported in HR patients [1, 32, 33], which support that these regions of the protein are important for PHEX function. The evidence suggests p.Gly649_Arg651del can be considered a likely pathogenic variant for HR disease (Additional file 1 Supplementary Table 3).

A second novel variant identified was a canonical splice donor site, c.54 + 1G > A, in intron 2 of the *DMP1* gene in patient 3 (Fig. 3b). This splice site variant was of particular interest because the segregation in trio 3 showed that this homozygous variant in the patient was transmitted from the consanguineous parents who were first cousins and carriers of c.54 + 1G > A (Fig. 2c). The disease and population databases have not reported this variant. Fifty ethnically matched (Malay) normal controls were screened for c.54 + 1G > A. No controls were homozygous for the variant and the heterozygous genotype was observed in 12 (24%) of the controls. (Additional file 1 Supplementary Fig. 1). *DMP1* is a recessive gene and LOF mutations of the gene cause ARHR disease. Therefore, only the homozygous genotype in patient 3 causes the disease phenotype and the heterozygous carriers are not affected. The c.54 + 1G > A variant occurs at a nucleotide position that is highly conserved and multiple splicing prediction programs predict this variant has a deleterious effect due to the loss of wildtype donor site (Additional file 1 Supplementary Table 3). According to ACMG, canonical splice-site variants result in a null effect, however, functional analysis is required to confirm the impact of variant. Therefore, the c.54 + 1G > A variant in the *DMP1* gene is interpreted as a variant with uncertain significance in relation to HR disease since functional study has not been performed and the criteria are not sufficient for assigning the pathogenicity (Additional file 1 Supplementary Table 3) [31].

## Discussion

HR is a rare genetic disease with genetic heterogeneity [34]. There has been scarce genetic confirmation of HR in Malaysia. The present study was established to determine if molecular diagnosis of three Malaysian HR patients could be achieved after screening the common genes for HR. Although clinical, radiographic, and laboratory data can help diagnose HR, genetic testing is more reliable for accurate diagnosis. All patients in this study showed typical clinical, radiographic and biochemical features of HR, with no family history of HR.

In the present study, two variants in *PHEX* and one variant in *DMP1* were identified using WES in three unrelated patients with HR from Malaysia. The two *PHEX* variants were de novo in the patients who were both females. This reflects previous findings of a higher rate of the *PHEX* de novo mutations in females than in males [35]. This is likely due to X-linked mutations occurring more frequently on the X chromosome in paternal germ cells [34]. The presence of de novo variants is one of the criteria to consider when establishing the pathogenicity of a variant [31, 36]. The confirmation of paternity testing in the two families with de novo *PHEX* mutations and the index patients being affected, provide strong criteria towards classifying the pathogenicity of these variants.

The de novo nonsense mutation in *PHEX* (c.871C > T, p.Arg291Ter) identified in this study has frequently been reported in HR patients from several studies of other populations including European, Chinese, and Japanese which may suggest that the nucleotides of the Arg291 residue is a mutation hot spot in *PHEX* and the present study confirms that p.Arg291Ter variant is a cause of HR disease in the Malay population. The patients with the p.Arg291Ter mutation in the previous studies had a similar phenotype to patient 1 including hypophosphatemia, bone deformities and radiological signs of rickets which are typical phenotype of HR [1, 32, 37–48].

The novel in-frame deletion identified in this study is located in exon 19 of *PHEX* (p.Gly649_Arg651del). PHEX is a member of the M13 family of membrane-bound zinc-metallopeptidases. There is a sequence motif (^642^ENXADXGG^649^) in exon19 of *PHEX* that is highly conserved in the members of the M13 family. A previous study in neutral endopeptidase (NEP), another member of the M13 family, has shown that this conserved motif plays an important role in zinc binding and catalysis activity [49]. The glycine 649 residue is located in the ^642^ENXADXGG^649^ motif, therefore, the deletion of this residue may interfere with zinc binding and catalytic activity of PHEX. A study by Kinoshita et al. [33] also revealed a missense mutation at this position (p.Gly649ASP) in three affected patients with HR from one family. In addition, Francis et al. [32] and Zheng et al. [1] reported a missense mutation at Arg 651 of PHEX (Arg651Pro) in two unrelated patients with HR. Although the prediction tools did not predict this deletion as disease-causing, the previous reports may indicate the importance of glycine and arginine residues at positions 649 and 651 of PHEX*,* respectively, and that these positions may be mutation hot spots.

The novel splicing variant in the *DMP1* gene (c.54 + 1G > A) was also detected with WES. Canonical eukaryotic splicing relies on four conserved nucleotides, i.e. the donor sequence GT at the 5′ end of the intron, and the acceptor sequence AG at the 3′ end [50]. The variant c.54 + 1G > A is located within the canonical site at the 5′ end of intron 2 of the *DMP1* gene. Mutations at the canonical splice sequences usually lead to single exon skipping [51]. The prediction of aberrant splicing and the involvement of highly conserved splice site sequences, provided evidence that the variant is likely causing HR in the patient. Of note, the same nucleotide position in *DMP1* but with different base change (c.54 + 1G > C) has been reported in a Chinese family with HR disease. Two patients of the family are homozygous and the healthy parents are carriers of the c.54 + 1G > C variant. Ni et al. showed that c.54 + 1G > C in the *DMP1* gene leads to the skipping of the whole exon 2 which then disrupts the reading frame of *DMP1* [52]. Furthermore, the c.54 + 1G > C variant has been reported in gnomAD in the heterozygous state with a minor allele frequency (MAF) < 0.1% (0.00039%).

Although this research presents novel findings from a unique ethnicity which is rarely studied, it also has several limitations. Small sample size is one of the limitations which is due to the rareness of HR disorder and small area of data collection. Therefore, statistical analysis regarding phenotype and genotype of patients could not be performed. Moreover, genetic analysis was carried out only for parent-child trios and multi-generational genetic investigation was not available. This is particularly important for the family in which the parents of an affected child were carriers of the variant. Another limitation is the absence of functional analysis to fully understand the impact of novel variants.

## Conclusions

We have identified candidate disease-causing variants in three Malaysian HR cases of Malay ethnicity. Two of the variants were novel and our data support that the *PHEX* novel variant is likely pathogenic. However, the evidence was not sufficient for the novel *DMP1* variant for assigning the pathogenicity and has been classified as a variant of uncertain significance. This study extends the variant spectrum in *PHEX* and *DMP1* and has shown the advantages of WES as an approach for the diagnosis of genetic diseases that are genetically heterogeneous. However, using conventional sequencing approaches to analyze the common genes prior to WES may identify the causative mutations in a proportion of patients and reduce the cost of genetic analysis. The findings of this study can be useful in genetic counseling and management of patients. Functional analysis of the identified variants is needed to verify the pathogenicity of these variants and will help to understand the underlying mechanisms of the disease and development of targeted therapies.

## Supplementary Information

**Additional file 1: Supplementary Table 1.** Thermal cycling for PCR amplification of *PHEX* (Exons 8 and 19) and *DMP1* (intron 2). A peqSTAR block thermal cycler (VWR, Radnor, PA, USA) was used. **Supplementary Table 2.** High resolution melting (HRM) analysis. Thermal cycling followed by Melting curve analysis was performed on a LightCycler 480 (Roche, Switzerland). **Supplementary Fig. 1.** Temperature shifted difference plots of patients and control group. **Supplementary Table 3.** Pathogenic prediction of the *PHEX* and *DMP1* variants. Different prediction software tools were used and the pathogenicity of the variants was assigned based on ACMG.

## Data Availability

The datasets used and analyzed during the current study are available from the corresponding authors on reasonable request.

## Abbreviations

HR: Hypophosphatemic rickets
PHEX: phosphate regulating gene with homologies to endopeptidases on the X chromosome
DMP1: dentin matrix acidic phosphoprotein 1
WES: Whole exome sequencing
XLHR: X-linked hypophosphatemic rickets
LOF: loss-of-function
HGMD: Human Gene Mutation Database
ADHR: autosomal dominant HR
ARHR: autosomal recessive HR
HHRH: hereditary HR with hypercalciuria
XRHR: X-linked recessive HR
FGF23: fibroblast growth factor 23
ENPP1: ectonucleotide pyrophosphatase/phosphodiesterase 1
SLC34A3: solute carrier family 34, member 3
CLCN5: chloride channel 5
1,25(OH)_2_D3: 1,25-dihydroxyvitamin D
ALP: alkaline phosphatase
PTH: parathyroid hormone
MREC: Medical Research and Ethics Committee
gnomAD: Genome Aggregation Database
ESP: Exome Sequencing Project
HSF: Human Splicing Finder.
