# Supplementary material for: Whole exome sequencing identifies two novel variants in PHEX and DMP1 in Malaysian children with hypophosphatemic rickets
Source: Ital J Pediatr. 2022 Dec 8;48:193. doi: 10.1186/s13052-022-01385-5 (PMC9730657; doi:10.1186/s13052-022-01385-5)
Supplement: Supplementary file 1 — Additional file 1: Supplementary Table 1. Thermal cycling for PCR amplification of PHEX (Exons 8 and 19) and DMP1 (intron 2). A peqSTAR block thermal cycler (VWR, Radnor, PA, USA) was used. Supplementary Table 2. High resolution melting (HRM) analysis. Thermal cycling followed by Melting curve analysis was performed on a LightCycler 480 (Roche, Switzerland). Supplementary Fig. 1. Temperature shifted difference plots of patients and control group. Supplementary Table 3. Pathogenic prediction of the PHEX and DMP1 variants. Different prediction software tools were used and the pathogenicity of the variants was assigned based on ACMG. [file 13052_2022_1385_MOESM1_ESM.docx]

| **Supplementary Table 1.** Thermal cycling for PCR amplification of *PHEX* (Exons 8 and 19) and *DMP1* (intron 2) | | | | | | | |
| --- | --- | --- | --- | --- | --- | --- | --- |
| Step | | Temperature | | Time | | Number of Cycles | |
| Initial Denaturation | | 95°C | | 10 min | | 1 cycle | |
| Denaturation | | 95°C | | 45 s | | 40 cycles | |
| Annealing | | 60°C | | 45 s | |  |  |
| Extension | | 72°C | | 45 s | |  |  |
| Final Extension | | 72°C | | 10 min | | 1 cycle | |
| Cooling | | 4°C | | indefinite | | 1 cycle | |
| Exons 8 and 19 of *PHEX* and intron 2 of *DMP1* were analyzed using previously published primers for these genes (Goji et al., 2006; Koshida et al., 2010). PCR analysis was performed in volume of 50 µL. A peqSTAR block thermal cycler (VWR, Radnor, PA, USA) was used. | | | | | | | |
| **Supplementary Table 2.** High resolution melting (HRM) analysis | | | | | | | |
| Step | | | Temperature | | Time | | Number of Cycles |
| Initial Denaturation | | | 95°C | | 10 min | | 1 cycle |
| Amplification | Denaturation | | 95°C | | 10 s | | 45 cycles |
|  | Annealing | | 60°C | | 15 s | |  |
|  | Extension | | 72°C | | 10 s | |  |
| Melting Curve | | | 95°C | | 1 min | | 1 cycle |
|  |  |  | 40°C | | 1 min | |  |
|  |  |  | 65°C - 95°C | | 25 acquisitions per every 1°C increment | |  |
| Cooling | | | 40°C | | 10 s | | 1 cycle |
| The PCR-HRM reactions (10 μL) were performed in triplicate containing 1X LightCycler 480 High Resolution Melting Master (Roche, Switzerland), 0.3µM of forward and reverse primers, 15ng of genomic DNA, 2mM MgCl_2_ and PCR-grade water. Samples were processed on a LightCycler 480 (Roche, Switzerland) for thermal cycling followed by Melting curve analysis. | | | | | | | |


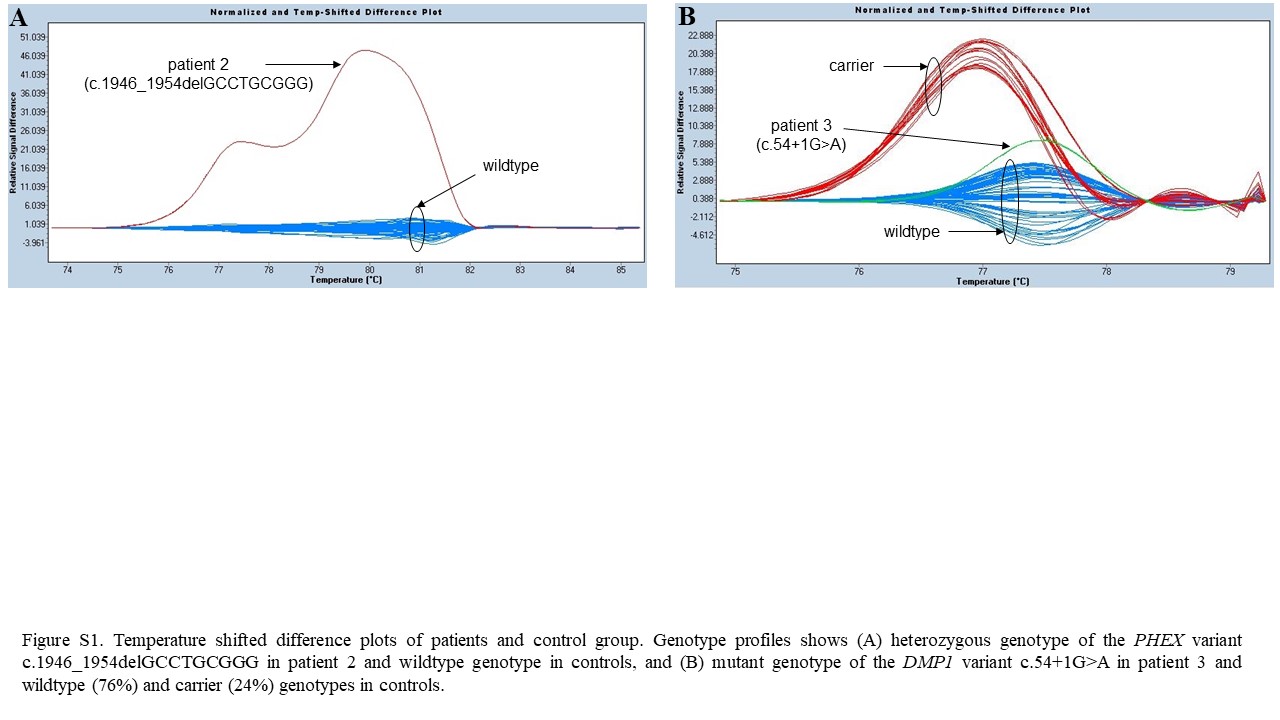


**Supplementary Figure 1.** Temperature shifted difference plots of patients and control group. Genotype profiles shows (A) heterozygous genotype of the *PHEX* variant c.1946_1954delGCCTGCGGG in patient 2 and wildtype genotype in controls, and (B) mutant genotype of the *DMP1* variant c.54+1G>A in patient 3 and wildtype (76%) and carrier (24%) genotypes in controls.

**Supplementary Table 3**. Pathogenic prediction of the *PHEX* and *DMP1* variants

| **Gene** | | *PHEX* | *DMP1* |
| --- | --- | --- | --- |
| **Variant** | | c.1946_1954delGCCTGCGGG | c.54+1G>A |
| **Protein consequences** | | p.Gly649_Arg651del | - |
| **Functional Consequences Prediction Score** | MutationTaster | N (0.9893) | D (1) |
|  | MutPred-Indel | 0.39 | - |
|  | HSF (Human Splice Finder 3.1) | - | Broken WT Donor Site (83.74 > 56.6 => -32.41%) |
|  | MaxEntScan (5'SS) | - | Broken WT Donor Site (9.09 > 0.91 => -89.99%) |
|  | SpliceAI | - | Donor loss (High precision) (0.97) |
|  | NNSPLICE 0.9 | - | Undetected 5' SS (0.97 > 0.00) |
|  | PhyloP | 5.115, -0.414, 1.67, 4.178, 0.521, 0.717, 5.115, 0.778, 3.553***** | 3.28 |
|  | GERP++ | 5.87, -2.6, 4.8, 5.87, 3.14, 3.01, 5.87, 1.85, 5.01* | 2.82 |
|  | PhastCons | 1, 1, 0.951, 0.997, 1, 1, 1, 1, 1***** | 0.99 |
| **Pedigree** | | 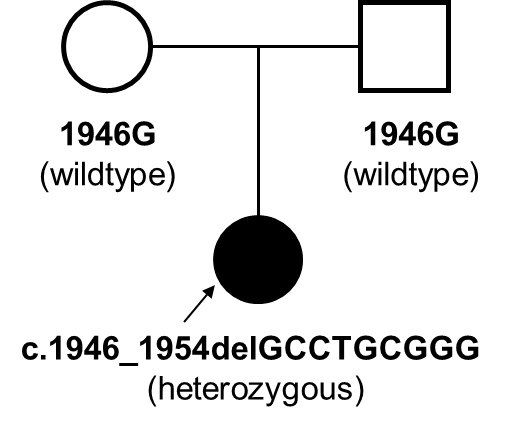 | 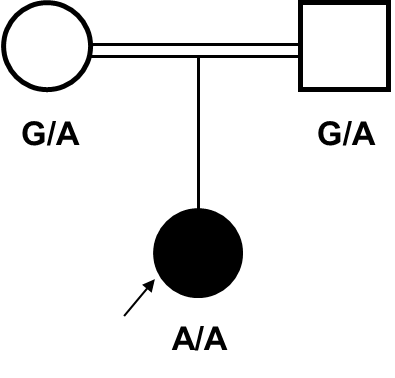 |
| **ACMG** | | LP (PS2, PM1, PM4) | Uncertain significance (PM2, PP3) |

MutationTaster: A = disease_causing_automatic, N = polymorphism, D = disease_causing;

MutPred-Indel: It returns a pathogenicity prediction score between zero and one, where variants with scores close to one are more likely to be pathogenic. If interpreted as a probability, a score threshold of 0.50 would suggest pathogenicity.

HSF (Human Splicing Finder): Scores with a range of 0–100, of which a higher score indicates strong or highly conserved splice site. The % represents the difference between the reference splice region and the splice region with the variation.

MaxEntScan: Scores with a range of 0–12, of which a higher score indicates strong or highly conserved splice site. The % represents the difference between the reference splice region and the splice region with the variation.

SpliceAI: The scores range from 0 to 1 and can be interpreted as the probability of the variant being splice-altering. 0.2 (high recall), 0.5 (recommended), and 0.8 (high precision) cutoffs.

NNSPLICE: Scores with a range of 0–1, of which a higher score indicates strong or highly conserved splice site. The % represents the difference between the reference splice region and the splice region with the variation.

PhyloP (phylogenetic P-values): The values vary between -14 and +6. Sites predicted to be conserved are assigned positive scores, while sites predicted to be fast-evolving are assigned negative scores.

GERP++ (Genomic Evolutionary Rate Profiling): The scores range from -12.3 to 6.17, with higher scores indicating higher evolutionary constraint. A score greater than 2 can be considered constrained.

PhastCons: The values vary between 0 and 1. The closer the value is to 1, the more probable the nucleotide is conserved.

ACMG (American College of Medical Genetics) criteria: P = pathogenic, L = likely pathogenic

*****The nine numbers correspond to the nine deleted nucleotides (c.1946_1954delGCCTGCGGG).

**References**

Goji, K., Ozaki, K., Sadewa, A.H., Nishio, H., and Matsuo, M. (2006). Somatic and germline mosaicism for a mutation of the PHEX gene can lead to genetic transmission of X-linked hypophosphatemic rickets that mimics an autosomal dominant trait. *Journal of Clinical Endocrinology and Metabolism* 91(2)**,** 365-370. doi: 10.1210/jc.2005-1776.

Koshida, R., Yamaguchi, H., Yamasaki, K., Tsuchimochi, W., Yonekawa, T., and Nakazato, M. (2010). A novel nonsense mutation in the DMP1 gene in a Japanese family with autosomal recessive hypophosphatemic rickets. *Journal of bone and mineral metabolism* 28(5)**,** 585-590. doi: 10.1007/s00774-010-0169-0.
